# Modification of Luffa Sponge for Enrichment of Phosphopeptides

**DOI:** 10.3390/ijms21010101

**Published:** 2019-12-22

**Authors:** Lili Dai, Zhe Sun, Ping Zhou

**Affiliations:** Key Laboratory of Analytical Chemistry for Biology and Medicine (Ministry of Education), College of Chemistry and Molecular Sciences, Wuhan University, Wuhan 430072, China; lilydai@whu.edu.cn (L.D.); 2014282030161@whu.edu.cn (Z.S.)

**Keywords:** luffa sponge, phosphopeptide, mass spectrometry, Matrix-assisted laser desorption ionization, solid-phase extraction

## Abstract

The enrichment technique is crucial to the comprehensive analysis of protein phosphorylation. In this work, a facile, green and efficient synthetic method was set up for quaternization of luffa sponge. The resultant luffa sponge showed strong anion-exchange characteristics and a high adsorption ability for phosphate ions. Along with the unique physical properties, e.g., tenacity and porous texture, quaternized luffa sponge was demonstrated to be a well-suited solid-phase extraction (SPE) material. The quaternized luffa sponge-based SPE method was simple, cost-effective and convenient in operation, and was successfully applied to the capture of phosphopeptides from protein digests. The enrichment approach exhibited exceptionally high selectivity, sensitivity and strong anti-interference ability. Four phosphopeptides were still detected by using the digest mixture of β-casein and bovine serum albumin with a molar ratio of 1:100. 21 phosphopeptides were identified from the tryptic digest of non-fat milk.

## 1. Introduction

Protein phosphorylation is a reversible posttranslational modification regulated by phosphatases and kinases. Abnormal protein phosphorylation events are often correlated with diseases [1,2]. Research of phosphopeptides provides valuable information to elucidate the biological regulatory mechanisms [3]. About 30% of cellular proteins would be phosphorylated during the physiological processes [4]. However, because of the dynamic and reversibility of phosphorylation, only 1–2% of the entire amount of protein is estimated to be phosphorylated at a specific moment [5]. In addition, the abundance of phosphopeptides received after digestion is much lower [6]. Matrix-assisted laser desorption ionization time-of-flight mass spectrometry (MALDI-TOF MS) is considered to be a powerful tool for the detection of phosphopeptides, however, of major concern is the poor ionization efficiency of phosphopeptides, caused by the low occupancy ratio of phosphopeptides and signal suppression of non-phosphopeptides. Therefore, prefractionation and the selective enrichment strategy of phosphopeptides is crucial for a comprehensive phosphoproteomics analysis [7].

Various methods have been extensively studied in recent decades, including chemical derivatization [8], immunoprecipitation [9], affinity chromatography with immobilized metal ions (IMAC) such as Fe^3+^ [10] and Ti^4+^ [11], affinity chromatography with metal oxides (MOAC) such as TiO_2_ [12] and ZrO_2_ [13], strong cation exchange (SCX) chromatography [14], strong anion exchange (SAX) chromatography [15], and hydrophilic interaction chromatography [16]. The effectiveness of a combined strategy, e.g., the use of SAX or SCX together with an affinity enrichment method (MOAC or IMAX), has also been demonstrated [17]. Owing to the existence of negatively charged phosphate groups (p*K*a = 1–2), the SAX materials would prefer to catch the phosphopeptides and screen the non-phosphopeptides via the charge difference between phosphopeptides and non-phosphopeptides [18]. SAX can not only enrich phosphopeptides but also fractionate phosphopeptides [19]. Amine-based materials are expected to provide a means for enriching phosphorylated proteins/peptides by SAX [20]. The electrostatic attraction and hydrogen bonding between amine and phosphate groups provide a foundation for selective enrichment [21]. Up to now, some amine-based materials have been used for selective enrichment of phosphopeptides, such as amino-functionalized materials [22], guanidyl-functionalized materials [23], polyethylenimine-functionalized materials [24], arginine-functionalized materials [25] and quaternary ammonium-functionalized materials [26,27]. Among them, quaternary ammonium materials, bearing permanent positive charges, provide the possibility of optimizing the best conditions for specific enrichment of phosphopeptides over a broad pH range.

Natural polymers have received increasing attention because they possess many virtues such as renewability, non-toxicity, inexpensiveness, biocompatibility and biodegradability. Luffa sponge, a natural macromolecular material, is obtained from the ripened dried fruit of *Luffa cylindrical*. Luffa sponge has a fibro-vascular reticulated structure with high porosity (79%–93%) and simultaneously low density (0.02–0.04 g/cm^3^) [28]. It is mainly composed of cellulose, hemicellulose and lignin, and may also contain a small amount of pectin, protein and trace elements such as calcium, magnesium, phosphorus, potassium and so on [29]. Luffa sponge exhibits excellent mechanical properties and a tough texture, which is acid and alkali resistant [30]. Owing to the abundance of active groups on the surface of luffa sponge, a variety of chemical modifications may be carried out to increase its functionality and the scope of its use [31,32]. Luffa sponge and its modified products have been used as sorbents for heavy metals [33], dyes [34] or phenols [35], biomatrixes for cell immobilization, and structural supports for biosorption in diverse biotechnological applications [36,37].

Luffa sponge has great potential to be used as a solid phase extraction material due to its physical and chemical properties. In this work, the strategy for quaternization of luffa sponge was investigated, and a new solid phase extraction approach based on quaternized luffa sponge was developed for selective enrichment of phosphopeptides. The performance of the enrichment approach was assessed.

## 2. Results and Discussion

### 2.1. Preparation and Characterization

The fiber of luffa sponge was principally made up of cellulose, hemicellulose and lignin. Small organic molecules were removed by methanol extraction. Alkali treatment had a beneficial effect on further modification. The general scheme for the preparation of quaternized luffa sponge A (QA) and quaternized luffa sponge B (QB) is presented in Scheme S1. The synthetic route to QA included three main steps. Firstly, epichlorohydrin (ECH) reacted with the hydroxyl groups of luffa cellulose to form epoxy cellulose ether. Then, the epoxide ring was opened by reacting with ethylenediamine. In the third step, triethylamine was connected to ethylenediamine moiety that had been grafted on the polymer chain to obtain QA. For the synthesis of QB, 3-chloro-2-hydroxypropyltrimethylammonium chloride (CHPTAC) was chosen as the etherification agent. Under alkaline conditions, CHPTAC was cyclized to epoxy propyl quaternary ammonium salt, and the latter reacted with cellulose through a nucleophilic ring opening reaction to generate cationic cellulose. To ensure QA and QB were carrying more quaternary amine groups, the reaction conditions such as feeding ratio, reaction time and temperature were optimized and has been explained in the experimental section. The obtained materials were characterized by scanning electron microscope (SEM) and X-ray photoelectron spectroscopy (XPS).

**Figure 1:**
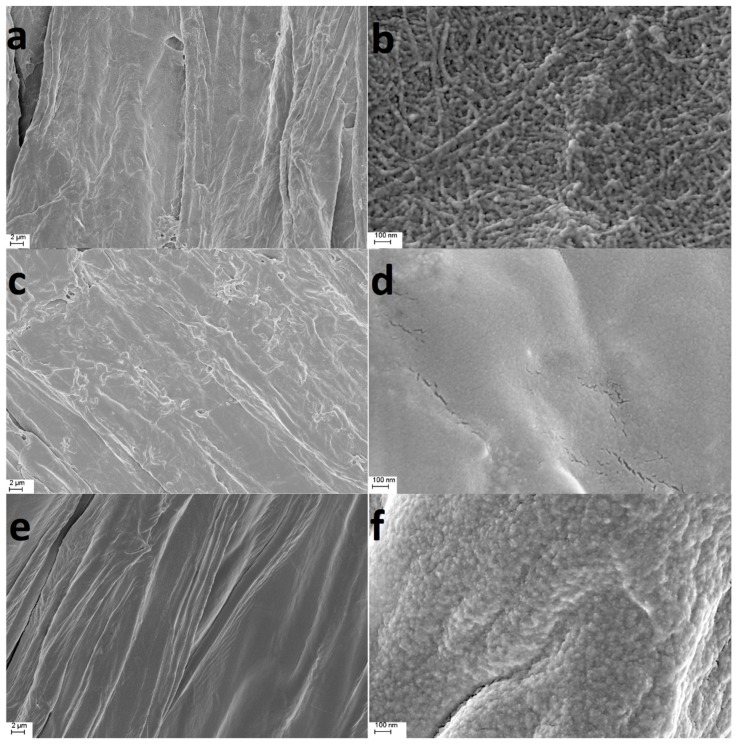
SEM images of (**a**,**b**) untreated luffa sponge, (**c**,**d**) modified luffa sponge QA, and (**e**,**f**) modified luffa sponge QB.

The differences in the morphology of untreated luffa sponge, QA and QB are displayed in Figure 1. The untreated luffa sponge material was composed of interwoven fibers finer than 100 nm. After modification, the microcosmic reticular structure on the surface of QA disappeared, implying that the fibers on the surface were partly impaired during the chemical modification process. QB had a smooth surface and was uniform in fiber microstructure.

**Figure 2:**
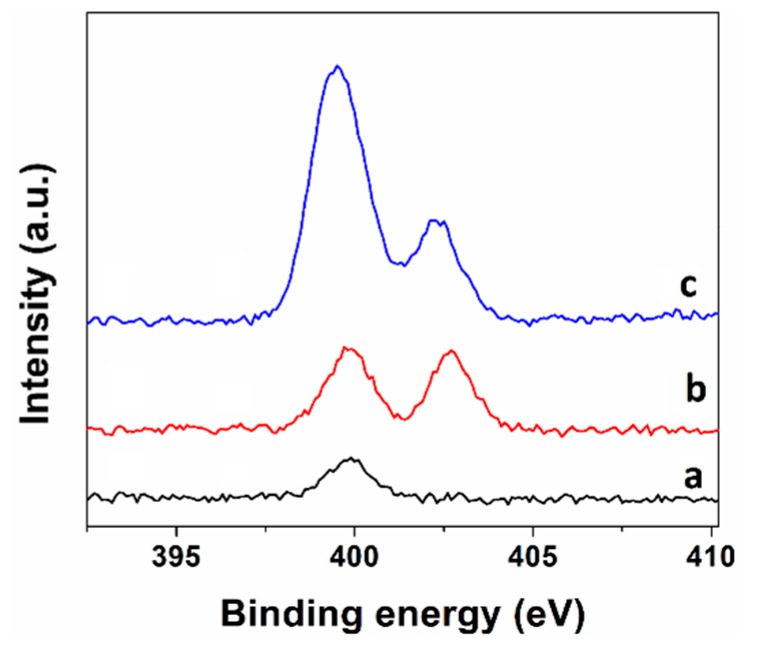
XPS spectra of N 1s of (**a**) untreated luffa sponge, (**b**) modified luffa sponge QB, and (**c**) modified luffa sponge QA.

The atomic percentages (C 1s, N 1s and O 1s) of untreated luffa sponge, QA and QB revealed by XPS are listed in Supplementary Table S1. The relative contents of N element on the surfaces of untreated luffa sponge, QA and QB were 0.67%, 5.60% and 2.56%, respectively, indicating that the ammonium groups were introduced to QA and QB. As shown in Figure 2, in comparison with untreated luffa sponge, both QA and QB displayed an additional peak at 402.5 eV in N 1s XPS high resolution spectra. This peak was attributable to the N element in quaternary ammonium groups, indicating that both synthesis routes were successful for the quaternization of luffa sponge.

### 2.2. Adsorption of Anions by Modified Luffa Sponge

**Figure 3:**
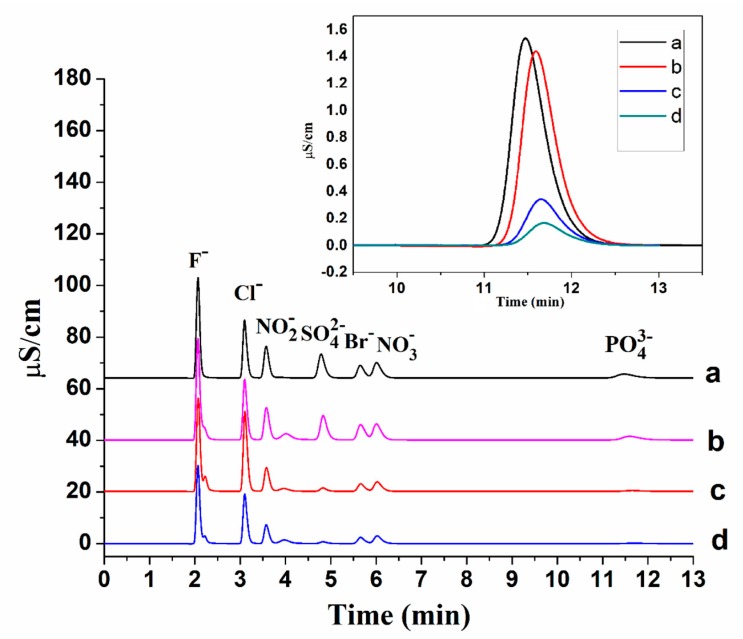
Ion exchange chromatograms of anions (**a**) before and after adsorption by (**b**) untreated luffa sponge, (**c**) QA, and (**d**) QB.

The adsorption performances of luffa sponge materials for inorganic anions were investigated. As shown in Figure 3, the untreated luffa sponge had almost no effect on adsorption of anions, however, both modified luffa sponge materials exhibited a significant effect, especially on sulfate and phosphate ions. 77.1% of phosphate ions were removed from the solution of mixed anions by QA, and 88.9% of phosphate ions were removed by QB. Although the total content of nitrogen element on the surface of QA revealed by XPS was higher than that of QB, the adsorption performance of QB was better, which was attributable to more quaternary ammonium groups on QB. Once the incubation temperature was set at 40 °C, 98.3% of phosphate ions were removed by QB.

### 2.3. Enrichment of Phosphopeptides by QB

**Figure 4:**
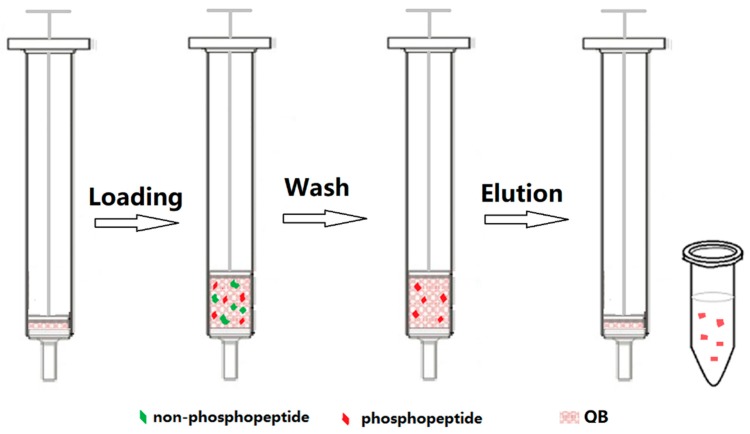
Schematic diagram of the procedure for enrichment of phosphopeptides with QB. Loading solution: 30 mM acetic acid-ammonium acetate in 20% acetonitrile, pH 4.0; washing solution: deionized water; elution solution: 5% trifluoroacetic acid.

Inspired by the aforementioned experimental results, we selected QB as a potential solid phase material for phosphopeptide enrichment. The schematic diagram of the procedure for solid phase extraction of phosphopeptides is shown in Figure 4. The enrichment mechanism was based on anion exchange. Phosphorylated peptides, carrying one or more phosphate groups, are more electronegative than other peptides in the same environment. When a proper loading solution was used, non-phosphopeptides would be exchanged by the anions in the solution while phosphopeptides could be retained on the solid phase. The pH of the loading solution was set at 4.0 to reduce the adsorption of non-phosphopeptides caused by electrostatic interaction. A small amount of acetonitrile was added to reduce the adsorption of non-phosphopeptides caused by hydrophobic interaction. Especially, owing to the characteristics of texture and fibro-vascular reticulated structure, the luffa sponge had open and free space for the exchange of matter, and was able to tolerate remarkable stresses and resume its original shape when brought again to rest. The extraction cartridge was assembled by packing QB within the barrel of a syringe between a sieve plate and the piston. The extraction was very convenient in operation. Since luffa sponge is almost an inexhaustible resource and the modification method for QB is facile and efficient, quaternized luffa sponge is low-cost.

**Figure 5:**
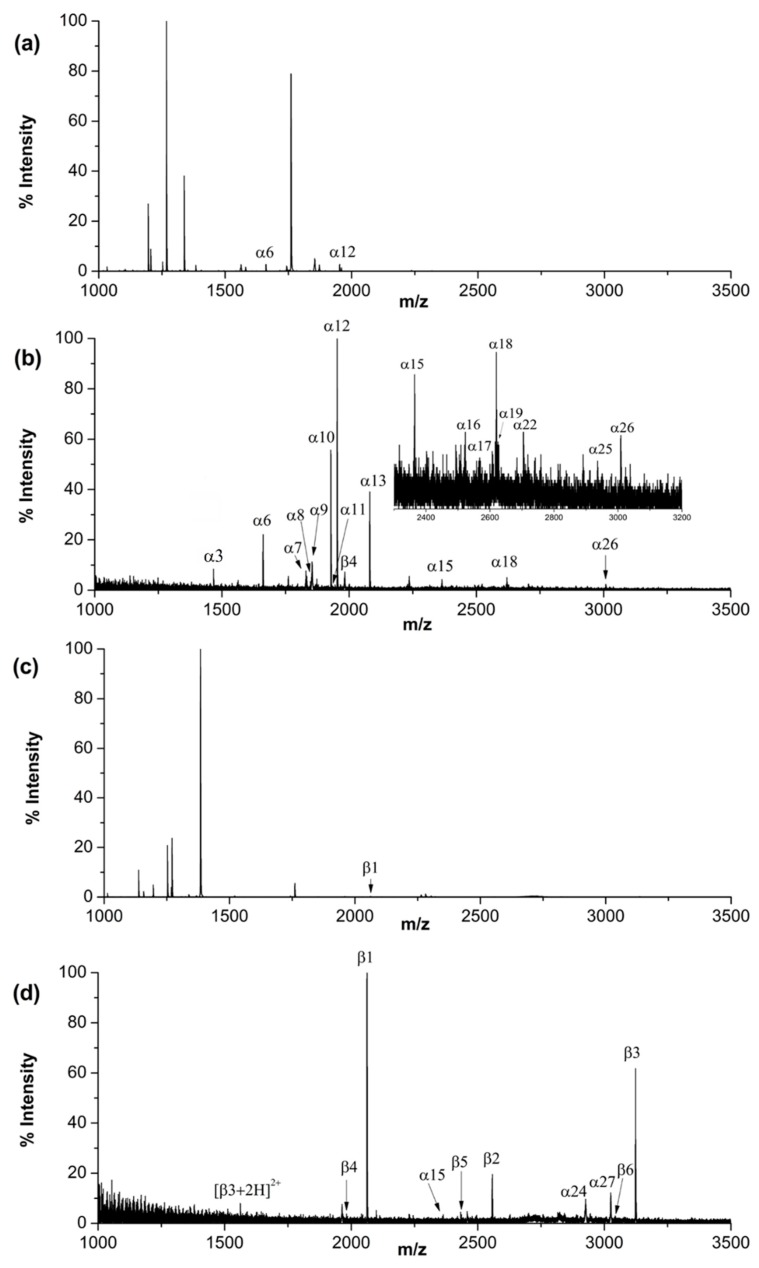
MALDI mass spectra of tryptic digests of α-casein (3 pmol) by direct analysis (**a**) or after enrichment using QB (**b**), and β-casein (3 pmol) by direct analysis (**c**) or after enrichment using QB (**d**).

The performance of QB for phosphopeptide enrichment was investigated by using tryptic digests of two typical phosphorylated proteins, namely α-casein and β-casein. When the tryptic digest of α-casein (3 pmol) or β-casein (3 pmol) was directly analyzed by MALDI-TOF MS, only weak signals of one or two phosphopeptides could be detected. After enrichment, the signals from phosphopeptides dominated the spectra. Taking α-casein digest as an example, the relative peak area of the phosphopeptides versus the total area of the peptide-ions in the mass spectrum was 69.2%. In contrast, the ratio *I*_phos_/(*I*_phos_ + *I*_non_) was 5.9% before enrichment. Eighteen phosphopeptides from α-casein digest (Figure 5b) and nine from β-casein digest (Figure 5d) were detected. The number of phosphopeptides enriched from α-casein was improved in comparison with the previously reported results enriched by commercial TiO_2_ [38,39], in which thirteen phosphopeptides were identified. The information about the phosphopeptides involved in this paper is displayed in Supplementary Table S2. 

To assess the selectivity of this enrichment approach to phosphopeptides, a non-phosphorylated protein, BSA, was chosen as a reference to examine the anti-interference ability. Enzymatic digest mixtures of β-casein and BSA with a molar ratio of 1:10 and 1:100 were tested. The MALDI-TOF MS spectra for the digest mixtures prior to enrichment are displayed in Supplementary Figure S1a,c. The majority of observed signals corresponded to non-phosphopeptides. After enrichment, peaks of phosphopeptides dominated in the mass spectra (Supplementary Figure S1b,d). When the molar ratio of β-casein to BSA was 1:100, four phosphopeptides could still be detected. Two among them were from α-casein due to protein impurities in β-casein. The results demonstrated the excellent selectivity of this method toward phosphopeptides in spite of the presence of a large amount of non-phosphopeptides.

To investigate the sensitivity of this approach, less amounts (300 fmol and 30 fmol) of β-casein digests were tested. As shown in Supplementary Figure S2, three phosphopeptides could still be detected even when the amount of β-casein was as low as 30 fmol.

### 2.4. Enrichment of Phosphopeptides from Non-Fat Milk

**Figure 6:**
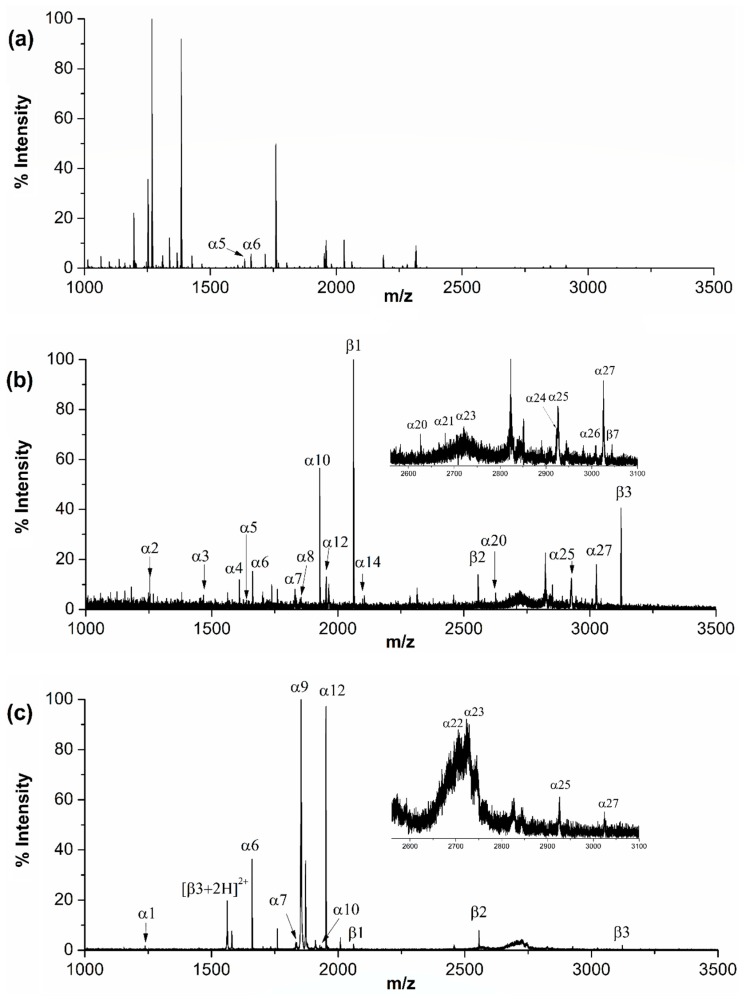
MALDI mass spectra of (**a**) tryptic digest of non-fat milk without any enrichment, (**b**) with QB enrichment, and (**c**) with TiO_2_ enrichment.

The application potential of QB to real samples was examined by selective enrichment of phosphopeptides from non-fat milk. Prior to enrichment, most peaks in the mass spectrum indeed corresponded to non-phosphopeptides in the tryptic digest (Figure 6a). After solid phase extraction using QB, three phosphopeptides from β-casein and eighteen phosphopeptides from α-casein were detected (Figure 6b). When the digest was enriched by TiO_2_ nanoparticles, only thirteen phosphopeptides in total were observed (Figure 6c). The number of phosphopeptides (21) captured from non-fat milk digest by QB also exceeded those captured by some other materials, such as mesoporous TiO_2_ nanoparticles (12) [40], Zr^4+^-immobilized magnetic covalent organic frameworks (14) [41], Ti(IV) and Nb(V) modified magnetic microspheres (19) [42], chitosan and polyethylenimine coated magnetic particles (17) [43] and magnetic guanidyl-functionalized metal–organic framework nanospheres (19) [44], and was comparable to that of titanium dioxide/ions on magnetic microspheres (23) [45]. The results demonstrated that the QB-based method exhibited good selectivity toward phosphopeptides in complex samples.

## 3. Materials and Methods

3-Chloro-2-hydroxypropyltrimethylammonium chloride aqueous solution (69 wt%) was purchased from Dibo Chemical Reagent (Shanghai, China) and used as etherifying reagents without further purification. Epichlorohydrin was purchased from Lingfeng Chemical Reagent (Shanghai, China). N, N-dimethylformamide (DMF), ethylenediamine, triethylamine, sodium hydroxide, methanol, ethanol, acetonitrile (ACN), isopropanol, urea, acetic acid, ammonium acetate and sodium bicarbonate were analytical grade and purchased from Sinopharm Chemical Reagent Co., Ltd. (Shanghai, China). The standard solutions of phosphate, nitrate, sulfate, nitrite, fluoride ion, chloride ion and bromine ion were purchased from the National Center for Analysis and Testing of Nonferrous Metals and Electronic Materials (Beijing, China). Iodoacetamide (IAA), 1,4-dithiothreitiol (DTT), trifluoroacetic acid (TFA) and 2,5-dihydroxybenzoic acid (DHB) were purchased from Aladdin Chemical Reagent Co., Ltd. (Shanghai, China). Bovine β-casein, bovine α-casein, trypsin (from porcine pancreas, TPCK-treated) and bovine serum albumin (BSA) were purchased from Sigma–Aldrich (St. Louis, MO, USA). The luffa sponge was purchased from Henan Ledu Pharmacy (Henan, China). Ultra-high molecular weight polyethylene sieve plate (UHMW-PE, diameter of 4.9 mm, thickness of 1.6 mm, and pore size of 20 μm) was purchased from Haohai Linfeng Technology Co., Ltd. (Wuhan, China). The syringe (1 mL) was purchased from Jinta Medical Equipment Co., Ltd. (Shanghai, China). Non-fat milk was purchased from a local supermarket.

### 3.1. Pretreatment of Luffa Sponge

The raw material of luffa sponge was peeled to remove the seeds and cut to small pieces. 10 g of luffa sponge was suspended in 300 mL of methanol in a 500 mL round-bottomed flask fitted with a condenser tube. The luffa sponge suspension was refluxed for 24 h at 60 °C. When dry, the luffa sponge was soaked in 5% (*w*/*w*) sodium hydroxide aqueous solution at room temperature for 24 h. The luffa sponge was then washed with deionized water, dried and sealed.

### 3.2. Synthesis of Quaternized Luffa Sponge

The first synthesis route was similar to that of Zhang et al. [46] whereby 1 g of pretreated luffa sponge was suspended in 40 mL of DMF and 20 mL of ECH in a 250-mL round-bottomed flask with a constant-temperature water bath at 85 °C and magnetic stirring. After 1 h, 2.6 mL of ethylenediamine was added dropwise into the flask. One hour later, 3.5 mL of triethylamine was added dropwise into the flask. Over a period of 60 min, the product was washed sequentially with deionized water and ethanol. When dry, the material was sealed for later use. The as-prepared material was coded as QA.

Quaternized luffa sponge was prepared by another route: 2 g of pretreated luffa sponge was soaked in 100 mL of sodium hydroxide aqueous solution (30%, *w*/*w*) overnight. After being washed with deionized water and dried, the luffa sponge was added to 80 mL of isopropanol in a flask with a constant-temperature water bath at 45 °C and magnetic stirring. Then 5 mL of sodium hydroxide aqueous solution (30%, *w*/*w*) was added and 7 g of CHPTAC aqueous solution was added dropwise into the flask. Over a period of 3 h, the product was washed sequentially with deionized water and ethanol. The material was dried and coded as QB.

### 3.3. Characterization of Quaternized Luffa Sponge Materials

The morphology of the materials was observed by a Sigma field emission scanning electron microscope (Zeiss, Germany). X-ray photoelectron spectroscopy measurement was performed on an ESCALAB 250Xi electron spectrometer (Thermo Scientific, Waltham, MA, USA) using a radiation source of Al Kα radiation with the energy of 1486.6 eV.

### 3.4. Adsorption of Anions

A mixture of anions containing F^−^, Cl^−^, NO_2_^−^, Br^-^, SO_4_^2−^, NO_3_^−^ and PO_4_^3−^ with the concentration of 10 mg/L each was made by diluting anion standard solutions with deionized water. 15 mg of untreated luffa sponge, QA or QB, was added to 1.5 mL of the solution of mixed anions. After being incubated at room temperature for 1 h, the suspension was centrifugated and the supernatant was collected for further ion chromatography analysis.

### 3.5. Ion Chromatography

An ICS 2500 ion chromatography system (Dionex, CA, USA) consisting of a GP50 gradient pump, AERS 500 suppressor, RFC-30 eluent generator and ED50 electrochemical detector was used in this study. IonPac^TM^ AS 11-HC (4 × 250 mm) and AG 11-HC guard (4 × 50 mm) columns (Dionex, CA, USA) packed with anion-exchange resin were used as the separation columns. The analysis was performed at 35 °C with the flow rate set at 1.5 mL/min in isocratic mode. The injection volume was 25 μL, and 36 mM potassium hydroxide was employed as mobile phase.

### 3.6. Peptide Sample Pretreatment

Bovine α-casein or bovine β-casein was originally made into stock solution at a concentration of 1 mg/mL with ammonium bicarbonate solution (50 mM, pH 8.0). Proteins were digested with trypsin by using an enzyme to substrate ratio of 1:50 (*w*/*w*), and the digestion was performed at 37 °C for 24 h.

BSA (1 mg) was dissolved in 100 μL of denaturing solution (8 M urea in 50 mM ammonium bicarbonate solution, pH 8.0). Then BSA was reduced by 10 mM DTT (final concentration) at 56 °C for 30 min. The reduced cysteine residues were alkylated with 20 mM IAA (final concentration) in the dark at room temperature for 30 min. The reduced and alkylated protein sample was diluted with 300 μL of 50 mM ammonium bicarbonate solution and then digested with trypsin at an enzyme to substrate ratio of 1:50 (*w*/*w*) by incubating at 37 °C for 24 h.

100 μL of non-fat milk was lyophilized to dryness and then denatured by adding 100 μL of denaturing solution (8 M urea in 50 mM ammonium bicarbonate solution, pH 8.0) at 56 °C for 30 min. The proteins were then reduced, alkylated, and digested the same way as BSA. All the tryptic digests were stored at −20 °C for future use.

### 3.7. Phosphopeptide Enrichment Procedure

The enrichment procedure involved three steps: load, wash and elution. One sieve plate was loaded into the bottom of a syringe barrel to construct a simple solid phase extraction device. Five milligrams of QB was put in between the sieve plate and the syringe piston. Ten microlitres of peptide mixture was added to 500 μL of loading solution (30 mM acetic acid-ammonium acetate buffer containing 20% ACN, pH 4.0). After being drawn in the syringe and incubated with QB for 1 h, the sample solution was pushed out. The QB material was then washed with loading solution and water in succession. The trapped peptides were eluted with 50 μL of 5% TFA.

The phosphopeptide enrichment by TiO_2_ was performed according to the literature [47]. TiO_2_ nanoparticles were prepared as previously described [48]. Before enrichment, 2 mg of TiO_2_ nanoparticles were dispersed in 500 μL of loading buffer (1 M glycolic acid, 5% TFA, 80% acetonitrile). Ten microlitres of protein digests were mixed with TiO_2_ suspension, and incubated for 1 h at room temperature, followed by centrifugation at 12,000g for 5 min. The supernatant was discarded and the remaining materials were washed sequentially with loading buffer and washing buffer (80% acetonitrile, 1% TFA). The phosphopeptides were eluted with 100 μL of NH_4_OH, pH 11 and the eluate was acidified with 10 μL of 100% formic acid. The acidified phosphopeptides were desalted on a Sep-Pak-C18 microcolumn. The purified phosphopeptides were eluted from the microcolumn directly onto the MALDI-target using DHB matrix (20 mg/mL DHB in 70% acetonitrile, 1% phosphoric acid and 0.1% TFA).

### 3.8. Mass Spectrometry Analysis

Peptides were analyzed using a 5800 MALDI-TOF/TOF mass spectrometry (AB SCIEX, Framingham, MA, USA) in positive ion mode. Matrix solution (mixture of 25 mg/mL DHB in 50% (*v*/*v*) ACN, 1% (*v*/*v*) phosphoric acid [49]) was mixed with equal volume of the eluate, from which 0.6 μL of solution was loaded onto the MALDI target. MS data were acquired in reflection mode. Four hundred shots were accumulated for each MS spectrum, and the data were processed by using Data Explore (AB SCIEX, Framingham, MA, USA).

## 4. Conclusions

In summary, we developed a feasible method for synthesizing quaternized luffa sponge and a new approach for the enrichment of phosphopeptides. Quaternized luffa sponge was demonstrated to be a well-suited solid phase extraction material. The quaternized luffa sponge-based extraction approach is simple, cost-effective, and convenient in operation. The enrichment approach exhibited exceptionally high selectivity and sensitivity toward phosphopeptides, and was successfully applied to the analysis of phosphorylated proteins in a complex sample. Due to the unique structural properties it bears, quaternized luffa sponge should be an attractive solid phase extraction material for a wide range of applications.

## Supplementary Materials

Supplementary Materials can be found at https://www.mdpi.com/1422-0067/21/1/101/s1.

## Abbreviations

MALDI	Matrix-assisted laser desorption ionization
TOF	Time-of-flight
MS	Mass spectrometry
SAX	Strong anion exchange
SPE	Solid-phase extraction
CHPTAC	3-Chloro-2-hydroxypropyltrimethylammonium chloride
ECH	Epichlorohydrin
IAA	Iodoacetamide
DTT	1,4-dithiothreitiol
BSA	Bovine serum albumin
