# Supplementary material for: Modification of Luffa Sponge for Enrichment of Phosphopeptides
Source: Int J Mol Sci. 2019 Dec 22;21(1):101. doi: 10.3390/ijms21010101 (PMC6982136; doi:10.3390/ijms21010101)
Supplement: Supplementary file 1 [file ijms-21-00101-s001.pdf]

Supporting information
for

Modification of luffa sponge for enrichment of phosphopeptides

Lili Dai, Zhe Sun, Ping Zhou*

Key Laboratory of Analytical Chemistry for Biology and Medicine (Ministry of Education), College of Chemistry and Molecular Sciences, Wuhan University, Wuhan, 430072, China

* Corresponding author: Prof. P. Zhou

E-mail: zbping@whu.edu.cn. (P. Zhou)

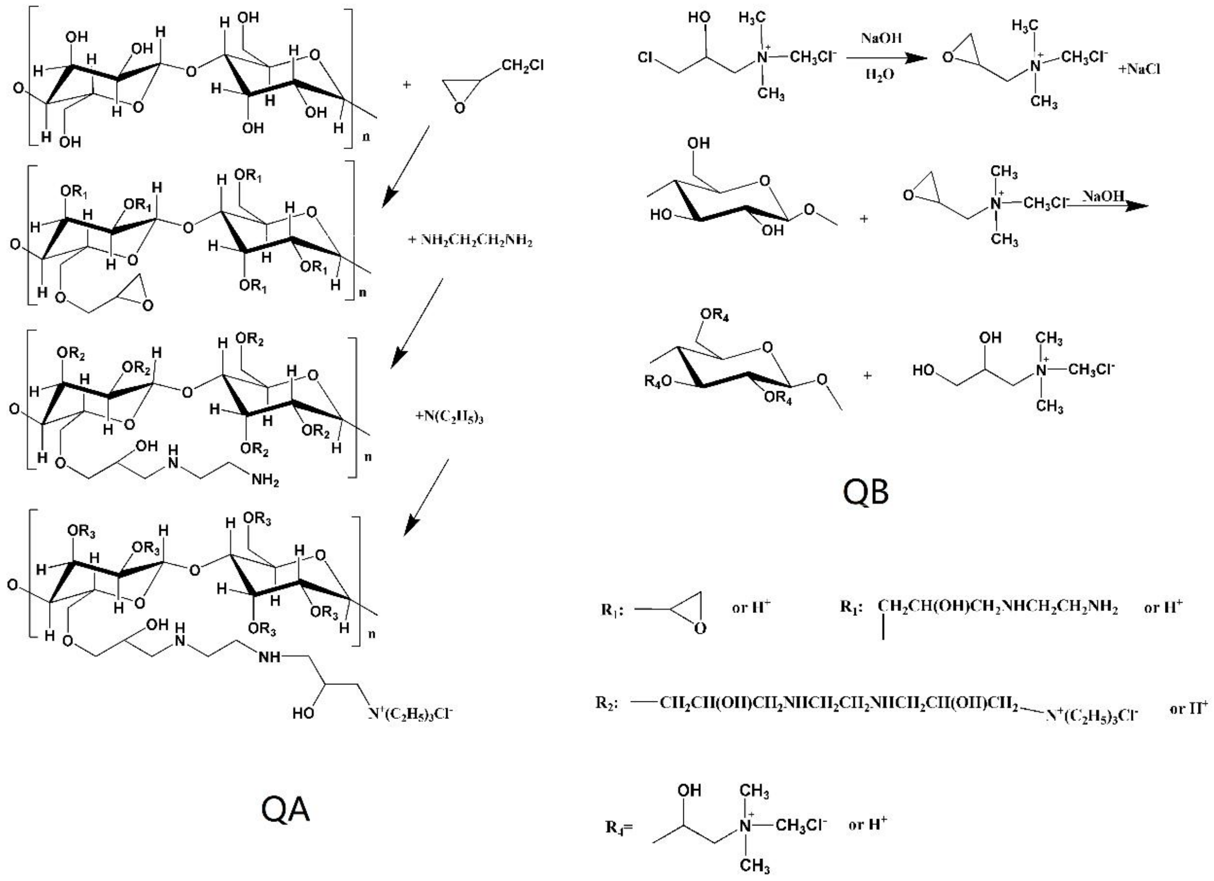

Scheme S1 Preparation of QA and QB.

Table S1 The atomic percentage (C1s, N1s and O1s) of untreated luffa sponge, QA and QB.

Name	C1s	N1s	O1s
Untreated luffa sponge	76.56%	0.67%	22.77%
QA	63.85%	5.60%	30.55%
QB	68.62%	2.56%	28.82%

Table S2 The information of the observed phosphopeptides obtained from tryptic digests of β-casein, α-casein, and non-fat milk.

No.	$[M+H]^+$ (monoisotopic)	No. of phosphorylation	Sequence
β1	2061.80	1	FQ[pS]EEQQQTEDELQDK
β2	2555.90	1	FQ[pS]EEQQQTEDELQDKIHPE
β3	3122.30	4	RELEELNVPGEIVE[pS]L[pS][pS][pS]EESITR
β4	1981.72	1	FQ[pS]EEQQQTEDELQDK
β5	2431.85		IEKFQ[pS]EEQQQTEDELQDK
β6	2966.89	4	ELEELNVPGEIVE[pS]L[pS][pS][pS]EESITR
β7	3042.36	3	RELEELNVPGEIVESL[pS][pS][pS]EESITR
α1	1253.53	1	TVDMME[pS]TEVF
α2	1466.58	1	TVDME[pS]TEVFTK
α3	1609.80	1	TVDM*E[pS]TEVFTKK
α4	1635.63	1	FFIF[pT]CLLAVALAK
α5	1660.97	1	VPQLEIVPN[pS]AEER
α6	1759.91	N	HQGLPQEVLNENLLR
α7	1832.77	1	YLGEYLIVPN[pS]AEER
α8	1847.62	1	DIGSE[pS]TEDQAMEDIK
α9	1854.89	1	YKVPQLEIVPN[pS]AEER
α10	1927.65	2	DIG[pS]E[pS]TEDQAMEDIK
α11	1943.61	2	DIG[pS]E[pS]TEDQAoMEDIK
α12	1951.91	1	YKVPQLEIVPN[pS]AEER
α13	2080.01	1	KKYKVPQLEIVPN[pS]AEERL
α14	2087.86	1	EVVG[pS]AEAGVDAASVSEEFR

α15	2361.80	1	PN[pS]VEQKHIQKEDVPSERY
α16	2518.92	1	VNEL[pS]KDIGSESTEDQAMEDIK
α17	2563.01	1	YKVPQLEIVPNSAEERLHSMK*
α18	2618.88	4	NTMEHV[pS][pS][pS]EE[pS]IISQETY K
α19	2622.95	4	pyroQMEAE[pS]I[pS][pS][pS]EEIVPN SVEQK
α20	2626.84	3	NANEEEYSIG[pS][pS][pS]EEAEVAT EEVK
α21	2679.11	4	VNEL[pS]KDIG[pS]E[pS]TEDQAME DIK
α22	2703.94	5	pyroQMEAE[pS]I[pS][pS][pS]EEIVPN [pS]VEAQK
α23	2719.39	5	QMEAE[pS]I[pS][pS][pS]EEIVPN[pS] VEQK
α24	2927.17	3	NANEEEYSIG[pS][pS][pS]EEAEVAT EEVK
α25	2936.18	3	EKVNEL[pS]KDIG[pS]E[pS]TEDQA MEDIKQ
α26	3008.01	4	NANEEEYSIG[pS][pS][pS]EE[pS]AEV ATEEVK
α27	3025.10	2	FPQ[pY]LQ[pY]LYQGPIVLNPWDQV KR

(*) methionine oxidation

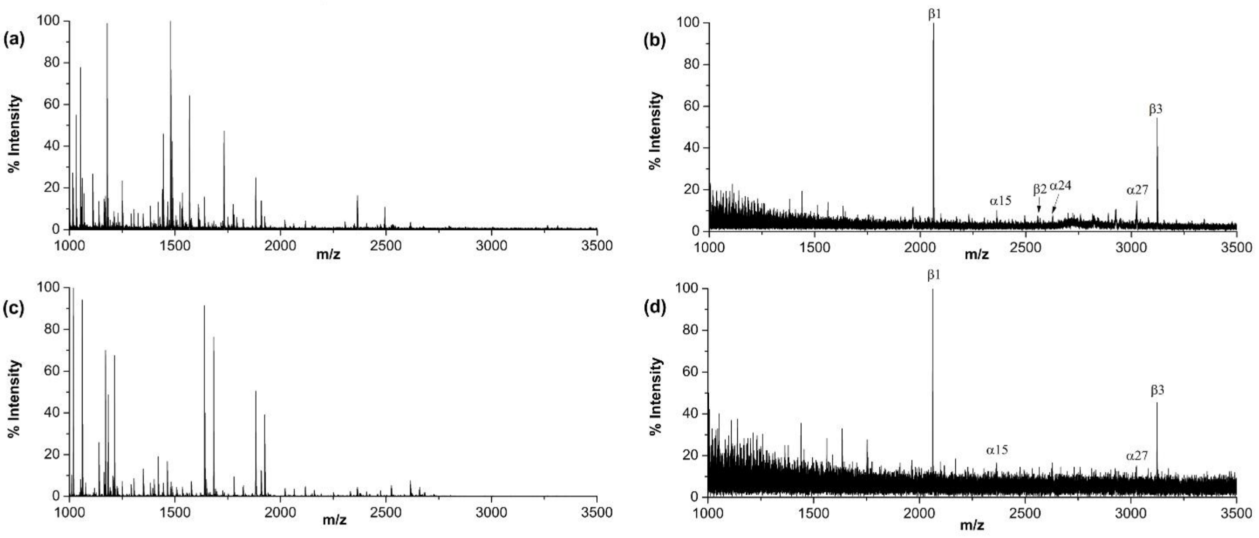

Fig. S1 MALDI mass spectra of the tryptic digest mixtures of β-casein and BSA (a, c) without or (b, d) with enrichment. The molar ratios of β-casein to BSA were (a, b) 1:10 and (c, d) 1:100, respectively. The concentration of β-casein was 3 pmol.

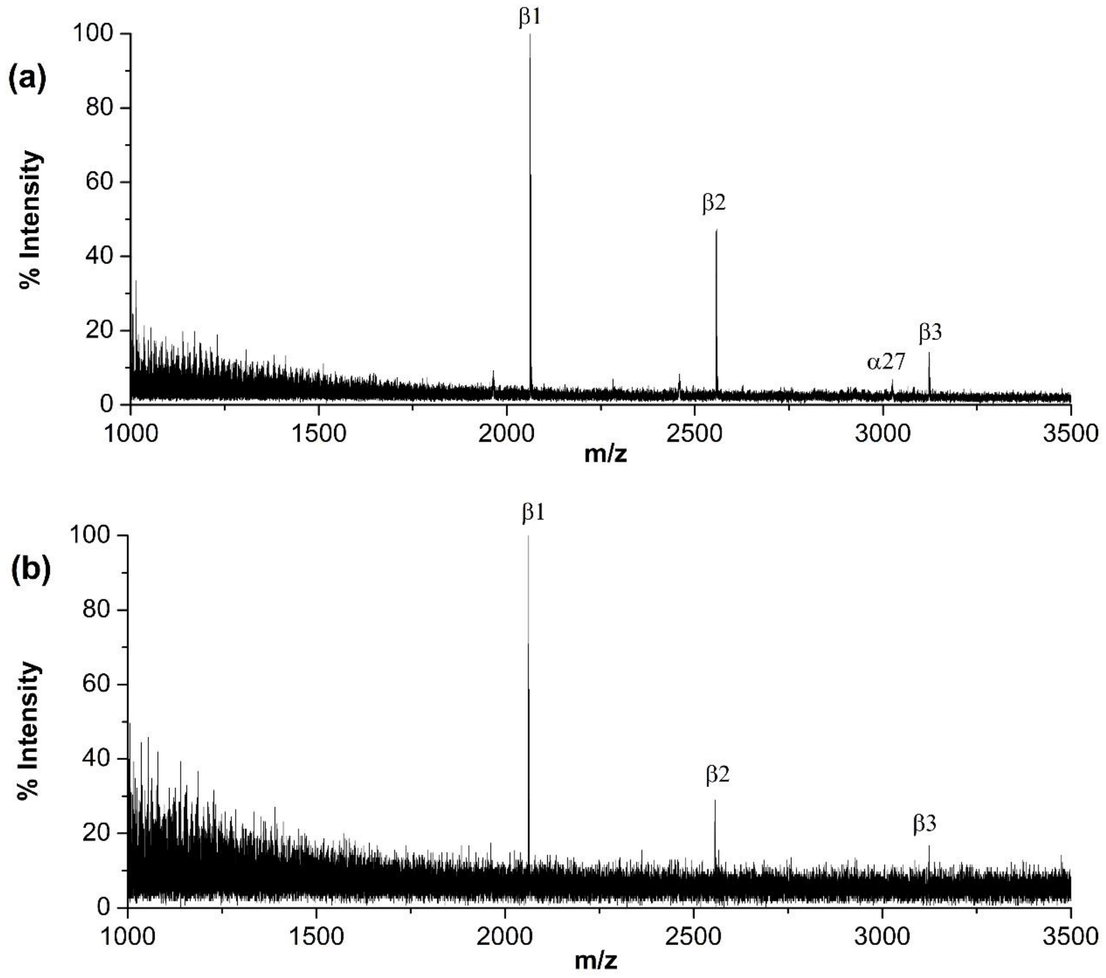

Fig. S2 MALDI mass spectra of tryptic digests of β-casein after enrichment using QB. The amounts of tryptic digest of β-casein used in the experiments were (a) 300 fmol and (b) 30 fmol.
